# Prognostic factors for corneal graft recovery after severe corneal graft rejection following penetrating keratoplasty

**DOI:** 10.1186/1471-2415-13-5

**Published:** 2013-02-25

**Authors:** Katsuya Yamazoe, Kyoko Yamazoe, Seika Shimazaki-Den, Jun Shimazaki

**Affiliations:** 1 Department of Ophthalmology, Tokyo Dental College, 5-11-13 Sugano, Ichikawa, Chiba, 272-8513, Japan; 2 Department of Ophthalmology, Keio University School of Medicine, Tokyo, Japan

**Keywords:** Penetrating keratoplasty, Rejection, Systemic steroid, Risk factor, Success rate

## Abstract

**Background:**

To investigate the outcome and prognostic factors for corneal graft recovery after severe corneal graft rejection following penetrating keratoplasty (PKP) treated with topical and systemic steroids.

**Methods:**

Fifty-eight eyes in 58 patients with severe corneal graft rejection following PKP were treated with topical and systemic steroids. Factors affecting the reversibility and maintenance of graft transparency were analyzed.

**Results:**

Graft transparency was restored in 37 of 58 eyes (63.8%). Clarity of the graft was maintained in 25 of 37 eyes after transparency was restored, while corneal decompensation developed at a mean of 6.0 ± 4.3 months in the remainder. The interval between rejection and treatment with systemic steroids was shorter in cases that recovered graft transparency (OR, 0.88, 95% CI. 0.80–0.97, *P* = 0.0093). Corneal decompensation after the recovery of corneal transparency tend to occur in cases of regraft (OR, 0.09, 95% CI. 0.01–0.54, *P* = 0.0091).

**Conclusions:**

Severe corneal graft rejection after PKP was reversible in approximately two-thirds of the cases, with graft transparency being maintained in two-thirds of them when treated with both topical and systemic steroids. Early treatment confers a benefit in terms of the recovery of graft transparency.

## Background

Corneal graft rejection is a severe complication that can follow penetrating keratoplasty (PKP), possibly leading to severe endothelial cell loss and corneal decompensation [1,2]. Risk factors for corneal graft rejection include donor age, graft size, repeated grafts, previous episodes of graft rejection, and corneal vascularisation [3-6]. Although the prognosis of corneal graft rejection has been reported [7-9], the severity of rejection varies and treatment has not been standardised in those studies. Graft rejection was treated with topical steroids with or without systemic steroids according to the severity of rejection or physicians choice [7-9]. Moreover, there is limited information on the influence of preoperative endothelial cell density before rejection on prognosis, and factors affecting corneal decompensation after the recovery of corneal transparency.

This study investigated the outcome in severe corneal graft rejection treated with a standardised protocol including topical and systemic steroids and factors affecting the reversibility and maintenance of graft transparency.

## Methods

### Patients and examination

We retrospectively studied 58 eyes in 58 patients who developed severe corneal graft rejection following PKP and were treated with topical and systemic steroids at Tokyo Dental College Ichikawa General Hospital between January 2005 and December 2010. Only patients with severe endothelial rejection defined as severe graft edema with an endothelial rejection line or five or more keratic precipitates appearing after attaining postoperative graft transparency were enrolled. All patients were hospitalised. Patients with a follow-up period of less than 6 months were excluded. Central corneal endothelial cell density (ECD) was measured using the EM-3000 (Tomey, Nagoya, Japan), and the value measured within 1 year before or after graft rejection were assessed. This study was conducted in accordance with the tenets of the Declaration of Helsinki and approval was obtained from ethics committees at our institution. All patients provided written informed consent for treatment with topical and systemic steroids.

### Treatment protocols

Postoperatively, the patients received medication with topical steroid to prevent graft rejection, according to the following protocol. Dexametasone phosphate 0.1% was administered five times daily and tapered over a period of 6 months, and fluorometholone 0.1% was continued unless infection or an uncontrollable increase in IOP occurred.

All patients diagnosed with graft rejection were treated with topical and systemic steroids. Dexamethasone phosphate 0.1% hourly was commenced immediately after diagnosis of a rejection and tapered according to the clinical response over several weeks. Treatment with systemic steroids was started in a few days after diagnosis of a rejection with intravenous dexamethasone phosphate or methylprednisolone. In the former, 8 mg/day dexamethasone phosphate was administered for 3 days, after which it was tapered to 6 mg/day for 3 days and 4 mg/day for 3 days. In the latter, 500 mg/day methylprednisolone was administered for 3 days, followed by oral 2 mg/day dexamethasone phosphate, which was then tapered over 2 weeks. Patients over 70 years old were given half of the steroid dose. Patients with a systemic disease, including infection, gastric ulcer and poorly controlled diabetes mellitus, were not treated with this protocol and not included in this study.

### Outcome measures

The main outcome measure was the rate of reversibility of corneal graft rejection. Rejection was considered reversible when the clinical signs had disappeared after treatment. The patients were divided into two groups: cases in which transparency was (Group 1) and was not (Group 2) restored. Parameters of interest included age, sex, diagnosis before PKP, type of surgery, history of glaucoma, previous episodes of rejection, ECD before corneal graft rejection, the interval between PKP and rejection, type of systemic steroid treatment and interval between rejection and treatment with systemic steroids. These factors were assessed and compared between Groups 1 and 2. In Group 1, the interval between treatment and the recovery of transparency, and whether transparency was maintained over the observation period, were also assessed. Cases in which transparency was maintained after recovery from rejection (Group 1A, n = 25) and cases wherein decompensation occurred after transparency was recovered (Group 1B, n = 12) were also compared.

### Statistical analysis

Statistically significant differences between the two groups were determined using logistic regression. A *P-*value of <0.01 was considered statistically significant. All statistical analyses were performed with SSRI software (SSRI, Tokyo, Japan).

## Results

### Demographics and clinical features

The patient demographics are shown in Table 1. The topical steroid that had been administered before rejection to prevent rejection was dexamethasone phosphate 0.1% in 25 cases, fluorometholone 0.1% in 19 cases and cyclosporine 0.1% in two cases. No steroid treatment had been administered before rejection in nine cases, including seven cases at a physician’s direction several years after surgery and two eyes against a physician’s recommendation. No data were available in three additional cases. The factor triggering rejection was related to broken sutures in five cases and self-discontinuation of topical steroid treatment in two cases. The mean interval between PKP and corneal graft rejection was 31.5 ± 36.7 months and that between corneal graft rejection and the administration of systemic steroids was 9.2 ± 9.7 days. All patients were treated for corneal graft rejection with above protocol, which is started with dexamethasone phosphate 0.1% eye drop and systemic steroids. The mean average follow-up after corneal graft rejection was 19.8 ± 12.7 months.

**Table 1:** Patient demographics

	**N**	**%**	**Mean ± SD**
Age (years)			62.7 ± 15.0
Sex			
Male	33	56.9	
Female	25	43.1	
Diagnosis before PKP			
Bullous keratopathy	21	36.2	
Regraft	14	24.1	
Corneal scar	13	22.4	
Corneal dystrophy	4	6.9	
Keratoconus	3	5.2	
Corneal ulcer	3	5.2	
Type of surgery			
PKP	43	74.1	
PKP + ECCE + IOL	13	22.4	
PKP + IOL suture	2	3.4	
history of glaucoma	17	29.3	
Previous episodes of rejection	8	13.8	
ECD before rejection			1401 ± 776
Interval between PKP and rejection (months)			31.5 ± 36.7
Interval between rejection and treatment with systemic steroids (days)			9.2 ± 9.7
Systemic steroid			
Dexamethasone phosphate	47	81.0	
Methylprednisolone	11	19.0	
Complications			
Elevated IOP	15	25.9	
Herpes simplex keratitis	3	5.2	
Fungal keratitis	1	1.7	

*ECCE*, extracapsular cataract extraction; *ECD*, endothelial cell density; *IOL*, intraocular lens; *IOP*, intraocular pressure; *PKP*, penetrating keratoplasty; *SD*, standard deviation.

### Outcome of steroid treatment

The mean best-corrected visual acuity (Log MAR) was 0.66 ± 0.75 before corneal graft rejection, 1.33 ± 0.87 before treating the corneal graft rejection and 1.03 ± 1.04 after treatment. Data on the ECD before rejection were obtained in 29 eyes (50.0%). In 18 of these eyes, data on the ECD after rejection were also obtained. The ECD in 18 eyes was 1556 ± 840 cells/mm^2^ before rejection versus 772 ± 464 cells/mm^2^ after rejection (*P* < 0.001). Graft transparency was restored in 37 of 58 eyes (63.8%). Nineteen of 41 cases that were observed for more than 1 year after rejection had a clear graft at 1 year and 4 of 7 cases that were observed for more than 3 years after rejection had a clear graft at 3 years. Graft clarity was maintained in 25 of 37 eyes (observation period, 20.7 ± 14.4 months), with corneal decompensation occurring 6.0 ± 4.3 months after the recovery of transparency in the remainder.

### Prognostic factors

Groups 1 and 2 are compared in Table 2. The interval between rejection and treatment was shorter in Group 1 (OR, 0.88, 95% CI, 0.80–0.97, *P* = 0.093). No differences were observed in age, sex, diagnosis before PKP, type of surgery, history of glaucoma, previous episodes of rejection, ECD before rejection, interval between PKP and rejection or treatment method.

**Table 2:** Comparison of the clinical characteristics between Group 1 (cases in which transparency was restored, n = 37) and Group 2 (cases in which transparency was not restored, n = 21)

	**Group 1**	**Group 2**	**OR**	***P-*value**
	**mean ± SD**	**mean ± SD**	**(95% CI)**	
	**n (%)**	**n (%)**		
Age (years)	59.4 ± 15.5	68.3 ± 12.5	0.95 (0.91–1.00)	0.037
Sex				
Male	23 (62.2)	10 (47.6)	1.81 (0.61–5.34)	0.28
Female	14 (37.8)	11 (52.4)	1.00	
Diagnosis before PKP				
Bullous keratopathy	13 (35.1)	8 (38.1)	0.88 (0.29–2.67)	0.82
Regraft	8 (21.6)	6 (28.6)	0.69 (0.20–2.36)	0.55
Corneal scar	9 (24.3)	4 (19.0)	1.37 (0.36–5.13)	0.64
Corneal dystrophy	3 (8.1)	1 (4.8)	1.76 (0.17–18.13)	0.63
Keratoconus	3 (8.1)	0 (0.0)	–	1.00
Corneal ulcer	1 (2.7)	2 (9.5)	0.26 (0.02–3.10)	0.29
Type of surgery				
PKP	27 (73.0)	16 (76.2)	0.84 (0.24–2.91)	0.79
PKP + ECCE + IOL	9 (24.3)	4 (19.0)	1.37 (0.36–5.13)	0.64
PKP + IOL suture	1 (2.7)	1 (4.8)	0.56 (0.03–9.37)	0.68
history of glaucoma	8 (21.6)	9 (42.9)	0.37 (0.11-1.18)	0.093
Previous episodes of rejection	4 (10.8)	4 (19.0)	0.51 (0.11–2.32)	0.38
ECD before rejection	1669 ± 747	806 ± 455	1.002 (1.001–1.004)	0.013
Interval between PKP and rejection (months)	33.3 ± 40.0	28.2 ± 30.7	1.00 (0.99–1.02)	0.61
Interval between rejection and treatment with systemic steroids (days)	6.2 ± 5.6	14.5 ± 12.8	0.88 (0.80–0.97)	**0.0093**
Systemic steroid				
Dexamethasone phosphate	29 (78.4)	18 (85.7)	0.60 (0.14–2.58)	0.50
Methylprednisolone	8 (21.6)	3 (14.3)	1.66 (0.39–7.07)	

*ECCE*, extracapsular cataract extraction; *ECD*, endothelial cell density; *IOL*, intraocular lens; *PKP*, penetrating keratoplasty; *SD*, standard deviation.

Groups 1A and 1B are compared in Table 3. Regarding the diagnosis before PKP, regraft was less prevalent in Group 1A (OR, 0.09, 95% CI, 0.01–0.54, *P* = 0.091). No differences were observed in age, sex, type of surgery, history of glaucoma, previous rejection episodes, ECD before rejection, interval between rejection and treatment, treatment or interval between treatment and recovery of transparency.

**Table 3:** Comparison of the clinical characteristics between Group 1A (cases in which transparency was maintained after recovery from rejection, n = 25) and Group 1B (cases in which the decompensation occurring after transparency recovered, n = 12)

	**Group 1A**	**Group 1B**	**OR**	***P-*Value**
	**mean ± SD**	**mean ± SD**	**(95% CI)**	
	**n (%)**	**n (%)**		
Age (years)	58.4 ± 17.4	61.5 ± 10.8	0.99 (0.94–1.03)	0.57
Sex				
Male	17 (68.0)	6 (50.0)	2.13 (0.52–8.70)	0.29
Female	8 (32.0)	6 (50.0)		
Diagnosis before previous PKP				
Bullous keratopathy	8 (32.0)	5 (41.7)	0.66 (0.16–2.73)	0.57
Regraft	2 (8.0)	6 (50.0)	0.09 (0.01–0.54)	**0.0091**
Corneal scarring	8 (32.0)	1 (8.3)	5.18 (0.57–47.32)	0.14
Corneal dystrophy	3 (12.0)	0 (0.0)	–	1.00
Keratoconus	3 (12.0)	0 (0.0)	–	1.00
Corneal ulcer	1 (4.0)	0 (0.0)	–	1.00
Type of surgery				
PKP	17 ((68.0)	10 (83.3)	0.43 (0.08–2.41)	0.33
PKP + ECCE + IOL	7 (28.0)	2 (16.7)	1.94 (0.34–11.20)	0.46
PKP + IOL suture	1 (4.0)	0 (0.0)	–	1.00
history of glaucoma	5 (20.0)	3 (25.0)	0.75 (0.15-3.84)	0.73
Previous episodes of rejection	1 (4.0)	4 (33.3)	0.13 (0.01–1.36)	0.09
ECD before rejection	1523 ± 666	2010 ± 876	0.999 (0.998–1.001)	0.19
Interval between PKP and rejection (months)	41.3 ± 44.2	16.8 ± 23.3	1.03 (0.99–1.06)	0.12
Interval between rejection and treatment with systemic steroids (days)	6.5 ± 5.5	5.8 ± 6.1	1.02 (0.89–1.15)	0.80
Systemic steroid				
Dexamethasone phosphate	20 (80.0)	9 (75.0)	1.33 (0.26–6.83)	0.73
Methylprednisolone	5 (20.0)	3 (25.0)	0.75 (0.15–3.84)	0.73
Interval between treatment and recovery of transparency (days)	72.5 ± 70.4	93.0 ± 208.0	0.999 (0.994–1.004)	0.65

*ECCE*, extracapsular cataract extraction; *ECD*, endothelial cell density; *IOL*, intraocular lens; *PKP*, penetrating keratoplasty; *SD*, standard deviation.

### Side effects of steroids

The side effects of topical and systemic steroids are shown in Table 1. Increased intraocular pressure (IOP), which was controlled with additional medication, was detected in 15 of 58 eyes (25.9%). Seven of the 15 patients had pre-existing glaucoma. Corneal infection developed within 3 months in 4 of 58 eyes (6.9%), including three cases of recurrent herpes simplex keratitis and one of fungal keratitis. No severe systemic side effects were observed and intravenous steroid administration was completed according to the protocol in all cases.

## Discussion

Severe endothelial rejection was treated with topical and systemic steroids in all cases and the rate of reversibility was 63.8%. The rate of reversibility was reported to be 51–63.3% in previous studies [7,8]. Wagoner *et al.* investigated 152 cases of severe endothelial rejection following PKP treated with topical steroids with or without systemic steroids and reported that graft survival was 42.6% at 1 year and 36.1% at 3 years [9]. Although our series was relatively small and some differences existed among other factors, the effectiveness of our treatment regimen in severe cases was comparable with that of earlier report. Our results suggest additional systemic steroids provide similar effective outcomes even in severe cases with severe edema. However, it is difficult to compare the status of patients before treatment with previous reports rigorously, and further study is needed. No serious systemic side effects were observed because we excluded patients in poor general health. Careful observation to detect elevated IOP and infection, especially herpes simplex or fungal keratitis, is necessary when treating patients with topical or systemic steroids.

In this study, a longer interval between corneal graft rejection and treatment with systemic steroids was associated with an increased risk of corneal decompensation after graft rejection. Risk factors for irreversibility after graft rejection reported in previous studies included donor age, patient age, diagnosis of BK, history of rejection or graft failure episodes [7,9]. Early treatment was reported to be associated with a better outcome [10] and our results support this finding. Factors affecting corneal decompensation after the recovery of corneal transparency were also investigated. Corneal decompensation occurs in one-third of the cases within approximately 6 months. A comparison of these cases with those in which corneal transparency was maintained revealed that regraft as a diagnosis before previous PKP was more frequent in the former. Notably, this factor was not associated with graft reversibility of transparency, and factor affecting graft reversibility of transparency was not associated with the maintenance of graft transparency. In regraft cases, more careful observation is needed after corneal transparency has been restored.

The endothelial cell density decreased significantly after corneal graft rejection. Musch *et al.* reported that the ECD decreased by 11.8% [11], while we observed a reduction in ECD in 18 cases in which ECD was determined before and after corneal graft rejection for a rate of 50.4%, which was much higher than that in Musch *et al.*[11]. Furthermore, cases in which ECD could be calculated, indicating the absence of severe edema, were believed to be mild cases compared with cases in which ECD could not be calculated in our study. Therefore, the endothelial cell loss may have been underestimated. These indicate the greater incidence of severe cases of rejection in our study compared with previously reported series [11]. Our results suggest the importance of preventing rejection, as well as close monitoring and appropriate and aggressive management of rejection when it occurs.

The interval between PKP and corneal graft rejection was 31.5 ± 36.7 months, which was longer than that in previous studies, including 15.4 ± 20.9, 10.4 ± 9.3 and 15.3 ± 14.4 months reported respectively by Epstein *et al.*[4], Naacke *et al.*[7] and Sangwan *et al.*[9]. One reason for this discrepancy between our results and those of these earlier studies may be that topical steroid treatment after PKP tended to be continued longer in our patients. In fact, we recently reported the efficacy of prolonged use of topical steroids for the prevention of rejection after PKP [12]. If no side effects are observed, such as elevated IOP, cataracts or infection, the long-term use of topical steroids is recommended.

## Conclusions

This study demonstrated that severe rejection was reversible in two-thirds of the cases reviewed, with graft transparency being maintained in two-thirds of them. A longer interval between corneal graft rejection and treatment was associated with an increased risk of corneal decompensation after graft rejection. Regraft as a diagnosis before previous PKP was associated with corneal decompensation after the recovery of corneal transparency.

## Competing interests

There are no competing interests for any authors for this manuscript.

## Authors’ contribution

KY, SS and JS contributed the study design, the data analysis, interpretation and manuscript writing. KY, KY and SS contributed ophthalmologic data collection. All authors read and approved the final manuscript.

## Pre-publication history

The pre-publication history for this paper can be accessed here:

http://www.biomedcentral.com/1471-2415/13/5/prepub
